# Approaching a discussion on the detachment of chlorpyrifos in contaminated water using different leaves and peels as bio adsorbents

**DOI:** 10.1038/s41598-023-38471-5

**Published:** 2023-07-11

**Authors:** Varsha Joshi, Manoj Kumar Jindal, Santosh Kumar Sar

**Affiliations:** 1 Department of Chemistry, Government V.Y.T. PG Autonomous College, Durg, Chhattisgarh India; 2 Department of Applied Chemistry, Bhilai Institute of Technology, Durg, 490001 India; 3 Present Address: Divecha Centre for Climate Change, Indian Institute of Science, Bangalore, India

**Keywords:** Environmental sciences, Environmental chemistry

## Abstract

The emerging contaminant chlorpyrifos, an insecticide, is generally used in agricultural fields to control termites, ants, and mosquitoes for the proper growth of feed and food crops. Chlorpyrifos reaches water sources for multiple reasons, and people who use water from nearby sources is exposed to chlorpyrifos. Due to its overuse in modern agriculture, the level of chlorpyrifos in water has drastically grown. The present study aims to address the problem arising from the utilization of chlorpyrifos-contaminated water. Natural bioadsorbents Bael, Cauliflower, Guava leaves Watermelon, and lemon peel were employed to remove chlorpyrifos from contaminated water under specific conditions of various factors, such as initial adsorbate concentration, dose of bioadsorbent, contact time, pH, and temperature. Maximum removal efficiency of 77% was obtained with lemon peel. The maximum adsorption capacity (qe) was 6.37 mg g^−1^. The kinetic experiments revealed that the pseudo second order model (R^2^ = 0.997) provided a better explanation of the mechanism of sorption. The isotherm showed that chlorpyrifos adsorbed in lemon peel in a monolayer and was best suited by the Langmuir model (R^2^ = 0.993). The adsorption process was exothermic and spontaneous, according to thermodynamic data.

## Introduction

Chlorpyrifos is an organic thiophosphate form of agrochemical insecticide^1^ and plays as EC 3.1.1.7 (acetylcholinesterase) and EC 3.1.1.8 (cholinesterase) inhibitor. It is an emerging environmentally noxious waste. USEPA^2^ included the chlorpyrifos compound in 400 listed products that can be advertised for many industrial, agricultural, and domestic pest control applications. In 2007, chlorpyrifos was recognized as the most widely used organophosphorus pesticide in the United States, with an annual use of around five million kg AI^3^. Eaton et al.^4^ reported that the annual global quantity of chlorpyrifos used between 2002 and 2006 was 25 million kg AI, with 98.5% of the quantity used in agricultural fields. The most intense quantities of chlorpyrifos are used in the crops cotton, corn, almonds, fruit trees, oranges, and apples. Gavrilescu^5^ and Van^6^ reported that pesticides are transported to aquatic systems and groundwater through irrigation and rainfall via runoff, drainage, and leaching^7^. Excess quantities of pesticides lead to contamination of the surrounding soil, and it is difficult to separate chlorpyrifos from soil. Therefore, instead of water-dissolved chlorpyrifos, soil-bound chlorpyrifos from eroding soil is more likely to be the cause of chlorpyrifos discharge into water from soil. Gebremariam et al.^8^ reported that pesticides are bonded to suspended sediments and particles and prevent leaching as soil strongly absorbs chlorpyrifos. However, it also creates a substantial off-cite migration pathway to runoff these pesticides in water bodies. Chlorpyrifos remains in the soil for approximately 60 to 120 days and then degrades into 3,5,6-trichloro-2-pyridinol, which is further broken down into organochlorine chemicals and carbon dioxide due to microbial activity^9,10^.

Chlorpyrifos has been used continuously at very high concentrations around the world, causing environmental elements to be contaminated in a number of ways. This has been substantiated by reports showing chlorpyrifos concentrations in groundwater, freshwater lakes, rivers, streams, city hurricane drains, rain, fog, marine sediments, sumps, sloughs, and even in air^11–19^. Additionally, significant health concerns have been found in both solid and liquid food samples from rural and urban areas. People and animals exposed to chlorpyrifos may suffer serious consequences, whether directly by inhalation or indirectly through skin, ocular contact and other sources such as dissolved water. According to Rahman et al.^20^, the insecticidal action of chlorpyrifos is based on its capacity to inhibit acetylcholinesterase activity (AChE). The synapse has an elevated concentration of acetylcholine (ACh) because of AChE inhibition, which overstimulates neuronal cells and induces muscle spasms. Chlorpyrifos metabolizes quickly and has a biological half-life of 15 to 30 h. According to Morgan et al.^21^, the major metabolite of chlorpyrifos in both the environment and the human body is 3,5,6-trichloro-2-pyridinol. In general, chlorpyrifos exposure in children can cause permanent neurodevelopmental abnormalities such as autism and attention deficit hyperactivity disorder (ADHD)^22^.

Many researchers have demonstrated empirical approaches adopted to remove or control specific pesticides or their combinations, such as ozonation with other oxidative processes, such as active carbon, UV radiation^23^, gamma radiation-based photolysis^24^, electrochemical treatment processes, combined UV Fenton processes^25^, oxidative degradation^26^, and nanomaterials^27^. In addition to employing activated biomass, modified magnetic activated biomass or nanocomposites have also been shown to be effective at removing organic toxicants as well as other elements, such as aluminum, uranium and others, from aqueous solution due to their reusability and large surface area^28–32^. Altintig et al. have shown that dyes can also be removed from water samples using modified magnetic activated carbon^33,34^.

Based on eco-friendly and economical aspects, remediation of pesticides via adsorption is the best approach via biowaste materials, which has been demonstrated to be effective in reaching the target of clean production. Research communities are continuously looking for creative, low-cost, effective, and environmentally friendly wastewater treatment and techniques that can be reused. Biosorption is one of the most prominent technologies with the best potential for removing pesticides from contaminated water. The concept of adsorption is old, but the use of waste materials for adsorption, which are readily available, affordable, and environmentally beneficial with any biological origin, is relatively new. Moreover, compared to liquid‒liquid extraction, solid phase extraction is more cost-effective and requires fewer operational steps for the extraction process^35^. In this context, the utility of various types of locally available biomass (solid phase) to extract chlorpyrifos from aqueous systems was investigated. Five vegetative adsorbates, namely, bael leaves, cauliflower leaves, guava leaves, lemon peel, and watermelon peel, were used, which have different carbon properties depending on the source. Lemon and watermelon fruit are a significant component of a healthy diet, with rich sources of nutrients, and provide health advantages^36^. These are intriguing sources of proteins, vitamins, carotenoids, minerals, essential oils, dietary fibers, and flavonoids^37^. These substances are abundant in functional groups such as amine (proteins), hydroxyl ion (cellulose), and carboxylic due to pectin^38^. Since time immemorial, the herbs bael, cauliflower and guava have been utilized in traditional Indian treatments, and these are connected to several significant medical properties. Their leaves have also been demonstrated to be used as bioadsorbents to remove toxic metal ions^39^, dyes^40^ and toxic chemicals^41^. In particular, lemon has been used worldwide during the COVID-19 pandemic as a source of vitamins to build the immune system.

This work aims to examine the use of these solid-phase bioadsorbents to extract chlorpyrifos from contaminated water bodies. In addition to performing thermodynamic and kinetic research to determine the adsorption behavior and the influencing mechanism, the experimental investigation included parametric experiments on adsorption with reference to pH, contact time, initial concentration of pesticide, dosage of bioadsorbents and thermal conditions. The loading capacity of the adsorbate was optimized, and its comparative selectivity toward chlorpyrifos removal efficiency in the presence of other analytes was investigated. Attempts have been made to decipher the factors responsible for the adsorption of chlorpyrifos via these five adsorbate materials.

This effort to show its practical utility for sustainability was used to remove chlorpyrifos from effluents from agricultural fields on a laboratory scale.

## Material and methodology

### Target material

Chlorpyrifos (make: Sigma‒Aldrich) was purchased from a chemical supplier. A 1 mg mL^−1^ stock solution was prepared with ethanol, and this solution was gently diluted with water to avoid any working error in standard solutions. Different concentrations of chlorpyrifos solutions ranging between 10 and 150 mg L^−1^ were prepared for laboratory batch-scale adsorption experiments.

### Selection of materials

India is the world's second largest producer of fruits and vegetables. Many fruits and vegetables are exported from India to other countries throughout the world. To consider in an economical manner, the selection of bioadsorbent can be linked with the production capacity and local availability of such material. In addition, natural bioadsorbents are always a better alternative than chemically created or modified bioadsorbents in terms of cost and environmental friendliness. Lemon is one of the fruit materials whose production rate has expanded dramatically over the last 3–5 years, as data indicate that production has increased by more than 50% from 2017 to 2020. In 2020, India ranked first in the world in lemon production, producing 3.72 MT lemon, accounting for 17.42% of the total global output, and it was commonly utilized during the COVID-19 epidemic because of its high vitamin C content. Watermelon is also one of the most abundant fruits in India, ranking third in the world with 2.79 MT produced. In terms of cauliflower output, India ranked second in the globe, producing 8.84 MT in 2020. Guavas are also among the top-producing goods in India. In India, Bael (*Aegle marmelos*) is commonly accessible^42^. Therefore, these five materials were chosen in this study to adsorb chlorpyrifos.

### Preparation of bioadsorbents

Lemon and watermelon were collected from a local fruit vendor, and the peel was removed and washed multiple times with distilled water. Peels may be classified as waste items, as all are being used after removing peels and discarding them into the waste bin because they are no longer important. Bael, cauliflower and guava leaves were also collected from the local area of Rajnandgaon district as huge quantity of these plants is accessible in Chhattisgarh state and washed properly as aforementioned. After washing, all the materials were cut into small segments to make it easier to devastate them. Then, these materials were exposed to sunlight for 2–3 days before being additionally oven dried for an additional 24 h at 90 °C to achieve a constant weight and to avoid any moisture content. The dried peels were crushed and passed through a sieve with a 125 µm size opening. The finished goods were stored in clean, airtight polyethylene bottles^43^.

### Instrumental characterization

Various instruments were used to analyze the materials, such as a Fourier transform infrared spectrophotometer (FTIR) in the region of 4000–400 cm^−1^ for chemical characterization of natural and pesticide-loaded biomass (Perkin Elmer, USA make, model: Spectrum IR Version 10.6.1), scanning electron microscope (SEM) (make: ZEISS, Germany, model: EVO series SEM EVO 18) and energy dispersive X-ray (EDX) (make: Oxford, UK, EDX system, model: INCA 250 EDS with X-MAX 20 mm) for surface morphology and elemental detection, pH meter (make: Orion Star, model: A325 digital) for pH adjustment, and magnetic stirrer (LabQuest MHPS5P) for stirring. A UV–Vis single beam spectrophotometer (make: Systronics, Switzerland, model: 117-Systronics) was used to analyze the concentration of chlorpyrifos.

### Quantity and quality assessment

Considering the quantity and quality aspects of the experiments, all glassware and chemicals were purchased certified with high standards. Each time, solution concentrations were determined before running the experiment and measured multiple times 3–4 to reconfirm the concentrations. All solutions were made only with double distilled water, and to avoid any contamination, glassware was washed with purchased cleaning agent and then with distilled water. All instruments were calibrated as per the standard process described by the manufacturers.

### Preparation and mechanism of chlorpyrifos

**Scheme 1:**
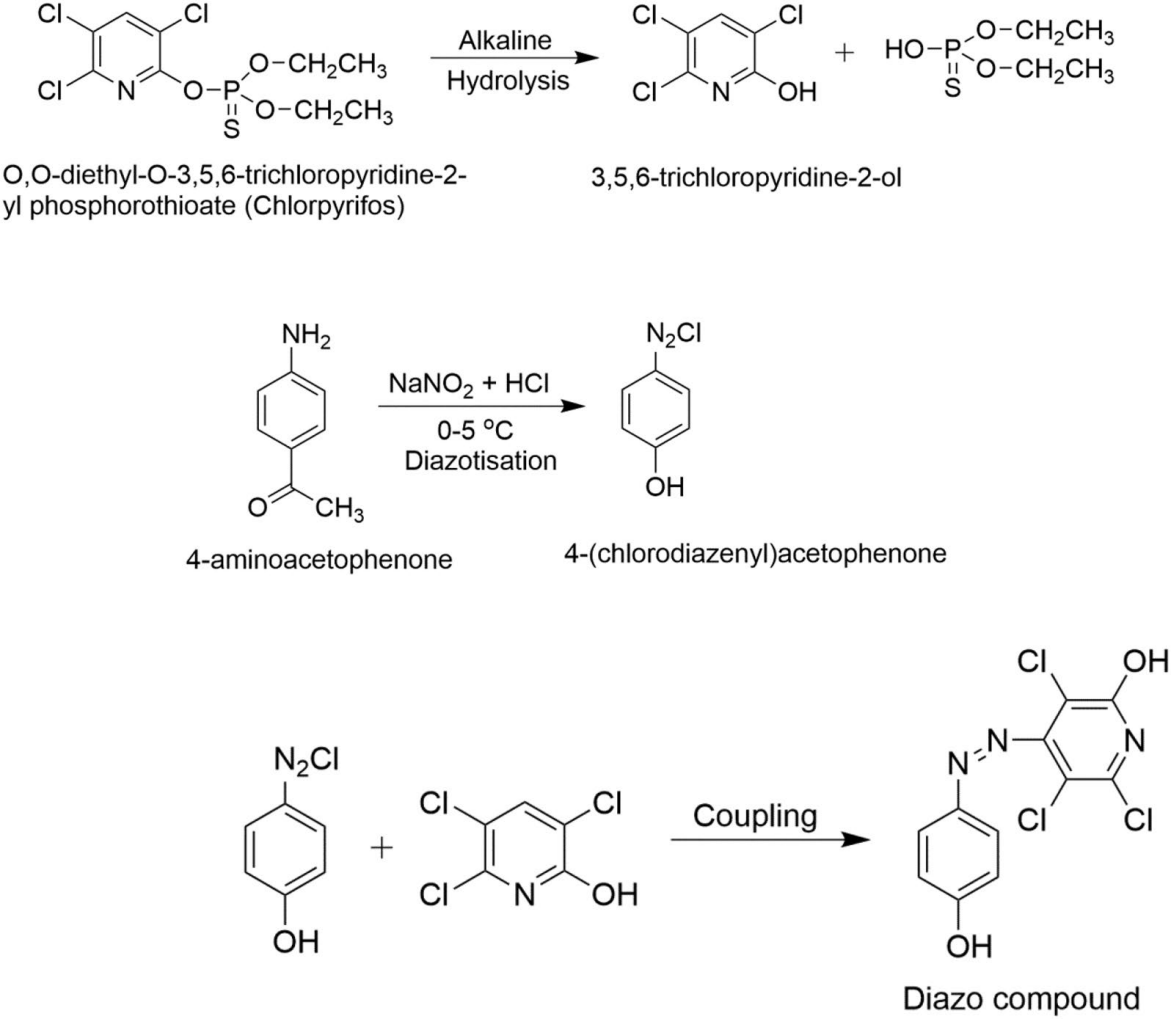
Schematic representation of the color reaction, which involves alkaline hydrolysis of chlorpyrifos followed by coupling of diazotized 4-(chlorodiazenyl) acetophenone with 1, 2, 4 trichloropyridin (TCP) to form orange red dye at 505 nm.

Different concentrations (1.2 to 10.5 g) of chlorpyrifos were taken in a 25 mL calibrated flask, and then 4.0 mL of 20% sodium hydroxide solution was added. The mixtures were heated for 5–10 min to finish the hydrolysis. After that, 1 mL of diazotized p-aminoacetophenone was added, the mixtures were thoroughly shaken, and then the color development process was performed, which took 10 min at 0 to 5 °C to develop the orange‒red color, as displayed in Scheme 1. The solution was then diluted to the proper concentration, and the absorbance of the blank and samples was measured at 505 nm^44^.

### Experimental procedure

Batch strategies have been applied to examine the adsorption of chlorpyrifos via the use of extraordinary bioadsorbent materials^45^. To begin, pesticide solutions with a concentration of 25 mg L^−1^ were prepared using the stock solution, and 0.5 g of bioadsorbent was added in a controlled environment. Under normal circumstances, the bioadsorbent solution was shaken for 60 min at 300 rpm in a shaker. After two hours, the sample was removed and centrifuged at 300 rpm for 10 min to separate all the undissolved particles. The solution, both with and without bioadsorbent, was then placed in a UV‒Vis spectrophotometer at 505 nm. Each experiment was carried out in a temperature-controlled setting in triplicate. Studies on chlorpyrifos sorption were carried out by modulating the contact period, initial concentration chlorpyrifos, pH of the solution, and dose of bioadsorbents^46^.

In each case, the percentage adsorption capacity was evaluated from Eq. (1).

1 $$\% \;{\text{Adsorption}} = \left[ {\frac{{C_{0} - C_{e} }}{{C_{0} }}} \right] \times 100$$

where C_o_ and C_e_ (mg L^−1^) signify the initial concentration and equilibrium concentration, respectively.

The maximum adsorption capacity (Q_e_) was computed through Eq. (2).

2 $$Q_{e} = \frac{{v(C_{0} - C_{e} )}}{w}$$

### Thermodynamic modeling

Equations (3) and (4) based on the Langmuir constant *K* were adopted to compute the enthalpy change (ΔH°), free energy change (ΔG°) and entropy change (ΔS°).

3 $$\Delta \,G^{{\text{o}}} = - RT\ln K_{eq}$$

where R represents the universal gas constant and T denotes the temperature.

4 $$\Delta \,G = \Delta \,H^{{\text{o}}} - T\Delta \,S^{{\text{o}}}$$

Rearranging Eqs. (3) and (4) to obtain Eq. (5).

5 $$\ln K_{eq} = \frac{{\Delta \,H^{{\text{o}}} }}{ - RT} - \frac{{T\Delta \,S^{{\text{o}}} }}{ - RT}$$

The linear form of Eq. (5) is stated as Eq. (6)

6 $$\ln K_{eq} = \left( {\frac{{\Delta \,S^{{\text{o}}} }}{R}} \right) - \left( {\frac{{\Delta \,H^{{\text{o}}} }}{R}} \right)\left( \frac{1}{T} \right)$$

### Kinetics modeling

Kinetics models were employed to decipher the adsorption rate and capability of the rate-controlling steps. In the prevailing work, the kinetic records obtained from experiments were analyzed using pseudo-first and second-order models. The equation of first order is usually expressed as follows.

7 $$\frac{dq}{{dt}} = k_{1} (qe - qt)$$

where qe and qt represent the mass of adsorbed chlorpyrifos in mg g^−1^ at equilibrium and time t (min), respectively, and k_1_ represents the rate constant of pseudo-first-order sorption (min^−1^). Equation (7) is linearized in the form of Eq. (8) as given below.

8 $$\ln (q_{e} - q_{1} ) = \ln q_{e} - k_{1} t$$

Equation (9) shows the pseudo-second-order kinetic rate equation.

9 $$\frac{dq}{{dt}} = k_{2} (q - q)^{2}$$

where K_2_ denotes the pseudo-second-order sorption rate constant (g mg^−1^ min^−1^). The linearized form of the above equation is expressed as.

10 $$\frac{t}{{q_{e} }} = \frac{1}{{k_{2} }}qe^{2} + \frac{1}{{q_{e} }}$$

### Adsorption isotherms

#### Langmuir isotherm model

The broadly adopted isotherm for modeling adsorption experiments is the Langmuir model, which is legitimate for monolayer sorption on surfaces with a defined range of equal sites and is represented by Eq. (11).

11 $$Q_{e} = \frac{{Q_{\max } .K_{L} .C_{e} }}{{1 + K_{L} C_{e} }}$$

where Q_e_ and K_L_ are Langmuir isotherm model parameters associated with most adsorption potential and free energy of adsorption, respectively. The linearized shape of the Langmuir equation can be written by Eq. (12).

12 $$\frac{{C_{e} }}{{Q_{e} }} = \frac{1}{{Q_{\max } .K_{L} }} + \frac{{C_{e} }}{{Q_{\max } }}$$

The Langmuir constants Q_e_ and K_L_ were computed by scheming 1/q_e_ as opposed to 1/C_e_.

The Langmuir isotherm equation, which has the following form, was used to calculate the unitless separation factor (R_L_).

13 $${\text{R}}_{{\text{L}}} = \frac{1}{1 + KL.Ci}$$

where Ci represents the initial chlorpyrifos concentration in mg L^−1^.

Adsorption is encouraged when the R_L_ value is between 0 and 1, but it is not encouraged when the R_L_ value is more than 1 or equal to zero.

#### Freundlich isotherm model

The Freundlich isotherm is an empirical Eq. (14) primarily based on heterogeneous sorption on a surface.

14 $$Q_{e} = K_{f} C_{e}^{1/n}$$

where K_f_ denotes the Freundlich constant that suggests adsorption potential and n denotes the adsorption intensity. The linearized shape of the Freundlich isotherm is represented by Eq. (15).

15 $$\log Q_{e} = \log K_{f} + \left( \frac{1}{n} \right)\log C_{e}$$

The values of K_f_ and *n* were calculated by plotting ln q_e_ vs. ln C_e_.

**Figure 1:**
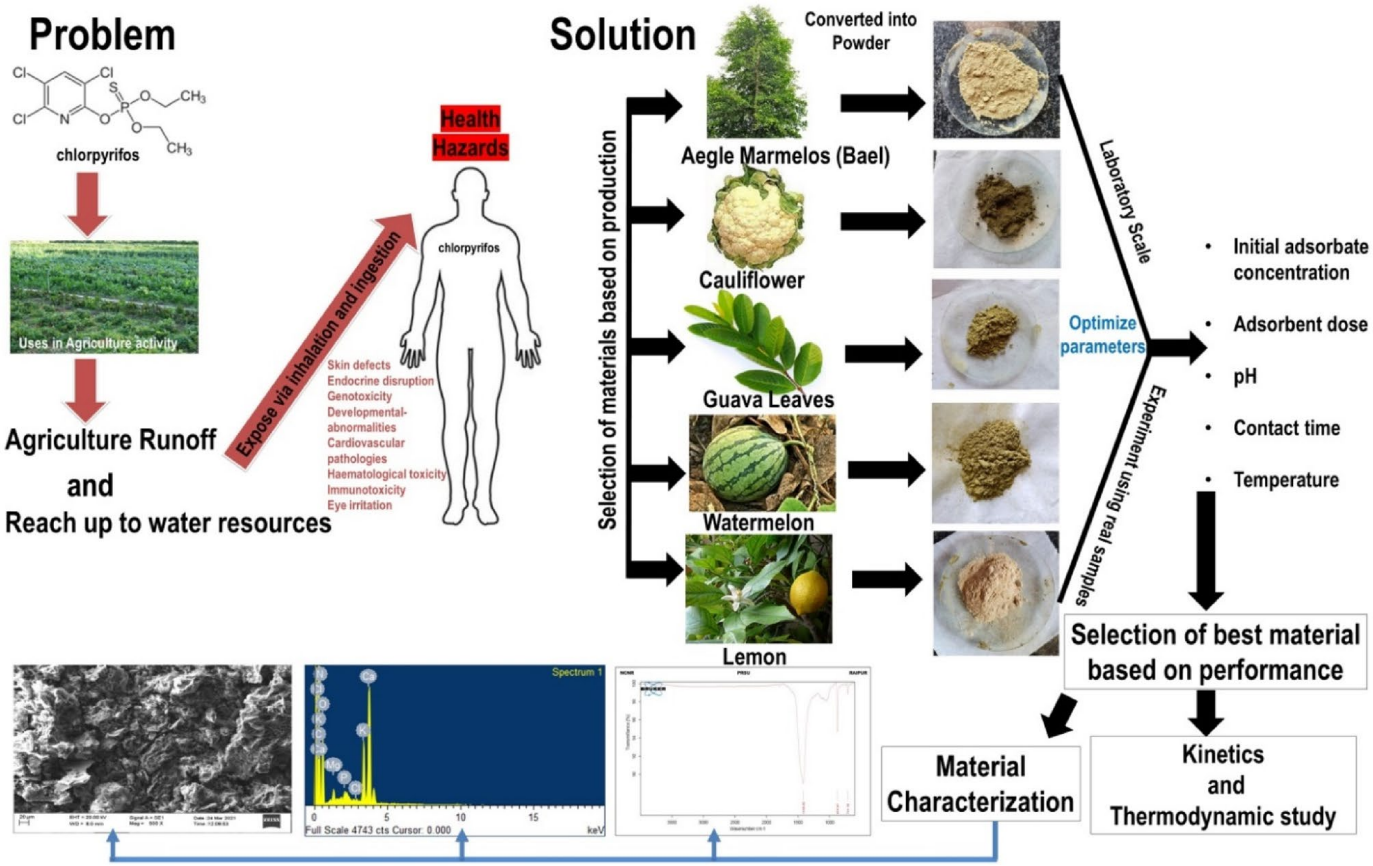
Steps of the problem-to-solution process that involves removing chlorpyrifos using various bioadsorbents.

Figure 1 depicts the overall problem-to-solution phases.

### Plant ethics statement

The plant collection and use was in accordance with all the relevant guidelines in the manuscript file.

## Results and discussion

### Adsorption as a function of bioadsorbent dosage

**Figure 2:**
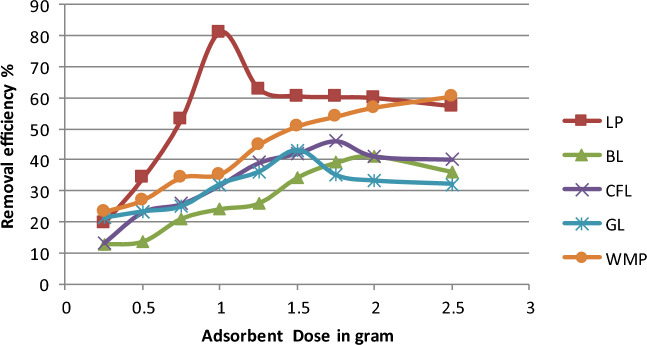
Variation in removal efficiency of chlorpyrifos with different bioadsorbents dosage (Initial concentration = 25 mg L^−1^, contact time = 60 min, pH 3 and temperature = 25 °C).

To investigate the impact of bioadsorbent dose, bioadsorbent dosages ranging from 0.25 to 2.50 g were used for all the bioadsorbents. The percent removal efficiency was plotted against bioadsorbent dose to evaluate the impact of bioadsorbent dose, which is shown in Fig. 2. A maximum removal percentage of adsorbate was observed with the lemon peel bioadsorbent at a 1 g dose. Bioadsorbents with an adsorption capacity of 0.25 g exhibited the least. The removal percentage of bioadsorbents increases as the dose increases because of many adsorption sites.

However, chlorpyrifos uptake steadily decreases due to two factors: first, many adsorption sites are available for the same quantity of chlorpyrifos, and second, a high quantity of bioadsorbents in a small amount of accessible space clumps together, limiting the diffusion and adsorption path. The observed sequence of chlorpyrifos removal efficiency was LP (lemon peel) > WMP (watermelon peel) > CFL (Cauliflower leaves) > GL (Guava leaves) > BL (Bael leaves).

### Function of contact time

**Figure 3:**
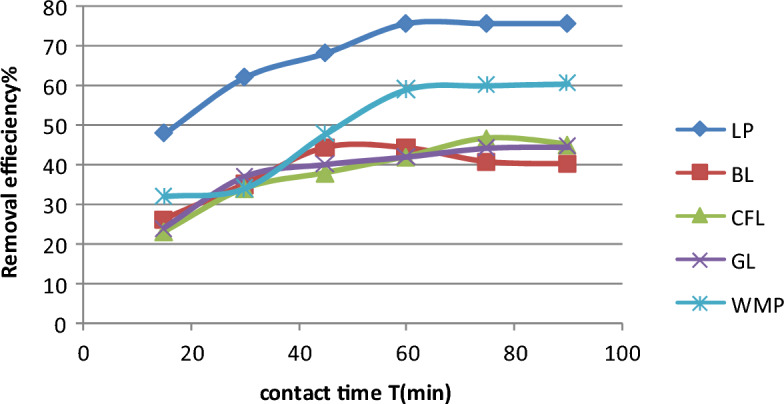
Variation in removal efficiency of chlorpyrifos with contact time (Initial concentration = 25 mg L^−1^, Bioadsorbent dose = 1 g, pH 3 and temperature = 25 °C).

Laboratory-scale batch experiments were performed in a series of flasks (conical) with a consistent bioadsorbent dose of 1 g in all samples to explain the influence of contact duration on chlorpyrifos (Co = 25 mg/L) adsorption. The flasks were shaken in a water bath shaker for 15, 30, 45, 60, and 75 min, keeping the pH constant. The results revealed that the adsorption of chlorpyrifos needed an equilibrium period of 60 min (1 h) for both lemon peel and watermelon peel. Lemon peel exhibits 80% adsorption with 1 gm of dosage, while watermelon peel exhibits 60% adsorption with the same dose. However, the findings revealed that the first 20 min were responsible for up to 50% of the overall quantity of chlorpyrifos absorption. Figure 3 shows the adsorption efficiency of chlorpyrifos with contact time. The presence of a considerable number of empty sites on the bioadsorbent could explain the higher sorption rate during the first period (first 20 min). The gradient concentration between the adsorbate in solution and on the bioadsorbent surface widened. As a result, chlorpyrifos sorption tends to increase in the early phases. As time passes, the concentration gradient narrows due to the build-up of chlorpyrifos particles in the unoccupied sites, resulting in a drop in the sorption rate at the larger phases from 65 to 80%. Another bioadsorbent used in the experiment failed to effectively adsorb chlorpyrifos.

### Function of pH of the solution

**Figure 4:**
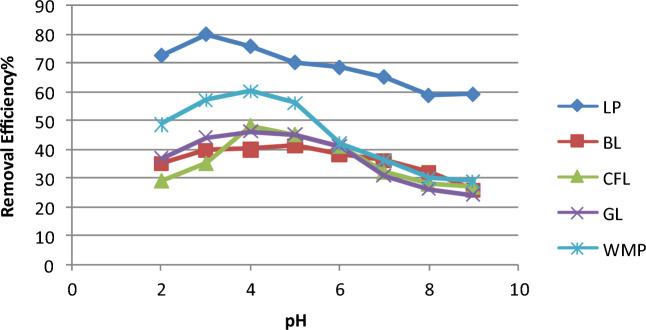
Variation in removal efficiency of chlorpyrifos with pH (Initial concentration = 25 mg L^−1^, Bioadsorbent dose = 1 g, contact time = 60 min and temperature = 25 °C).

The impact of pH on chlorpyrifos adsorption was also studied at 25 °C for initial concentrations of 25 mg L^−1^ chlorpyrifos solution adjusted with either 0.1 M NaOH or 0.1 M HCl at diverse pH values in the range of 2–10 in a series of polypropylene bottles. Each of the aforementioned bioadsorbents removed chlorpyrifos at different proportions depending on the solution pH, as shown in Fig. 4. Chlorpyrifos absorption improves gradually with increasing pH, attaining the highest value at pH 2–3. After that, the uptake of chlorpyrifos decreases with increasing pH up to 10. Furthermore, a rise in pH reduces chlorpyrifos uptake. The quantity of chlorpyrifos adsorbed is lowest at pH 8, and the application of chlorpyrifos further decreases. pH also affects the degree of ionization of chlorpyrifos in the adsorption medium. At high pH values, the amount adsorbed decreases in this investigation. Above this pH, chlorpyrifos is projected to become a negatively charged ion, resulting in reduced adsorption owing to repulsion between the anionic ion and surface layer. An acid solution was used to achieve a low pH value. It raised the quantity of protons in the solution, which vied for phosphate sites, causing the adsorption to slow down at very low pH ~ 2.

### Function of initial concentration

**Figure 5:**
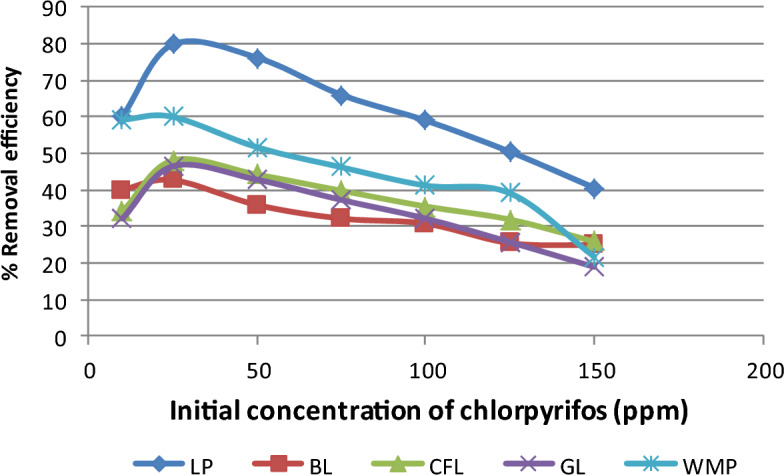
Variation in removal efficiency of chlorpyrifos with initial concentration of adsorbate (Bioadsorbent dose = 1 g, contact time = 60 min, pH = 3 and temperature = 25 °C).

Experiments were performed with various ranges of chlorpyrifos concentrations, such as 10, 25, 50, 75, 100, 125, and 150 mg L^−1^ at a fixed bioadsorbent dose of 1 g to evaluate the influence of efficiency on the initial chlorpyrifos concentration, as presented in Fig. 5. The graphical value with an initial chlorpyrifos concentration of 25 mg L^−1^ suggests that 80% elimination is possible, and equilibrium is achieved in an hour. The concentration gradients increased with adsorbate lemon peel in solution, which may explain the greater sorption rate during the initial period. Due to the build-up of phenol particles in the unoccupied sites, the concentration gradient between adsorbate lemon peel in solution and on the bioadsorbent surface decreases as time passes, resulting in a drop in the sorption rate at the larger stages between 25 and 60 min.

### Function of temperature

**Figure 6:**
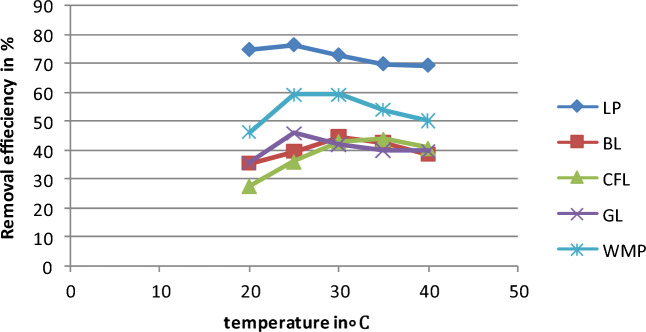
Variation in removal efficiency of chlorpyrifos with temperature (Initial concentration = 25 mg L^−1^, Bioadsorbent dose = 1 g, contact time = 60 min and pH 3).

Chlorpyrifos solutions of 25 mg L^−1^ concentrations with various biosorbents were prepared to investigate the influence of temperature on the removal process. All sample solutions were maintained at a pH of 3, and 1 g bioadsorbent was added to each. Then, the samples were agitated for 60 min at temperatures of 20, 25, 30, 35, and 40 °C (Fig. 6). Based on the findings, a rise in temperature only resulted in a minor change in adsorption capacity for lemon peel. In comparison to lemon peel, the minimum removal efficiency of the remaining four bioadsorbents ranged from 35 to 45%. For several bioadsorbents, the removal effectiveness was observed between 25 and 35 °C among the different studied temperatures. The adsorption of chlorpyrifos on the lemon peel bioadsorbent decreased as the temperature increased. The exothermic character of adsorption is supported by this diminishing tendency.

### Application in real samples

All the factors that influence adsorption were optimized, such as bioadsorbent dosage, contact time, pH of the solution, initial concentration of chlorpyrifos and temperature. After optimizing all the factors, optimized conditions were used on real samples collected from environmental and agricultural fields from different locations, as shown in Table 1. Chlorpyrifos (CPF) was detected in agricultural runoff water in a fairly wide range of distributions ranging from approximately 120–160 square kilometers in the Rajnandgaon area of Chhattisgarh state. Based on the wide use of this pesticide in this region, it was found that the chlorpyrifos value ranged between 2.97 and 6.43 µg/100 mL in real samples, which is higher than its acceptable limit (3 µg/100 mL). It appeared to be a persistent pesticide that showed genotoxicity and mutagenicity according to prior studies^4^. These data showed that the chlorpyrifos concentration was higher than the permissible limit in Rajnandgaon District. Water samples of agricultural runoff from Rajnandgaon district were collected, and their chlorpyrifos contents were fed into batch removal adsorption through five bioadsorbents, namely, lemon peel, watermelon peel, bael leaves, cauliflower leaves and guava leaves, and their adsorption efficiencies were calculated. Field application of the bioadsorbents to study their absorptive efficiency revealed that chlorpyrifos can be decontaminated using bioadsorbents prepared from lemon peel more efficiently than using five bioadsorbents, lemon peel, watermelon peel, bael leaves, cauliflower leaves and guava leaves, with maximum removal efficiencies ranging between 68 and 74% w/W.

**Table 1:** Various real samples from environmental and agricultural fields and their adsorption efficiency with different bioadsorbents.

Sampled zone	Type of environmental matrix*	Chlorpyrifos conc. µg/100 mL (Range)	Cauliflower leaves % w/W	Watermelon peel % w/W	Bael leaves % w/W	Lemon peel % w/W	Guava leaves % w/W
Rajnandgaon	Agricultural runoff	5.23–5.65	54	61	53	71	45
Dongargaon	Agricultural runoff	4.37–4.86	56	65	52	69	34
Gunderdehi	Agricultural runoff	2.97–3.24	52	65	43	73	45
Semerbandha	Agricultural runoff	5.34–6.43	48	56	46	68	61
Manipur	Agricultural runoff	5.28–5.64	44	67	41	74	57
Rajnandgaon	Agricultural runoff	4.67–5.09	49	62	46	77	54
Kaurikasa	Agricultural runoff	5.55–6.07	43	59	44	73	48
Bodal	Agricultural runoff	4.09–4.48	51	61	37	71	36
Boerdih	Agricultural runoff	3.98–4.02	50	54	40	69	37
Mohra	Agricultural runoff	5.48–6.24	46	65	41	74	44

Chlorpyrifos can enter the human body by inhalation and ingestion during agricultural activities and water intake. This may also have an impact on birds and animals via land, water, and air. Skin problems, genotoxicity, endocrine disruption, developmental abnormalities, cardiovascular diseases, hematological toxicity, immunotoxicity, and eye irritation are all potential effects on animals, humans, and birds^20^. Even tiny amounts of chlorpyrifos, such as 0.01 pounds per acre, can cause mortality in many aquatic creatures^47^. The neurotoxicity caused by CPF exposure significantly affects the health of children, and because of this consequence, several environmental regulatory bodies have long debated prohibiting the use of this chemical. Liu et al.^48^ studied 40 patients who had chlorpyrifos exposure at Chang Gung Memorial Hospital between 2008 and 2017. According to one study, 70% of CPF is taken orally, while just 3% is absorbed through the skin in exposed persons. Rahman et al.^20^ revealed the probable health hazards of CPF, as it is a commonly used pesticide and is inexpensive and extremely efficient in controlling insects and providing greater yields. One disadvantage in India and other developing nations is that because this product is widely accessible and unbanned in such countries, it might reach stressed people and be used as a suicide material. According to research conducted in India, 40.5% of suicide attempts involve pesticides, and CPF was obtained in the blood of 131 people who died^49^. Controlling such chemical exposure is critical to meeting sustainable objectives and making the environment safe due to its dangerous effect. This work demonstrates the use of local materials to reduce the toxic impact of CPF in water sources.

### Kinetics study for the chlorpyrifos bioadsorbent system

**Figure 7:**
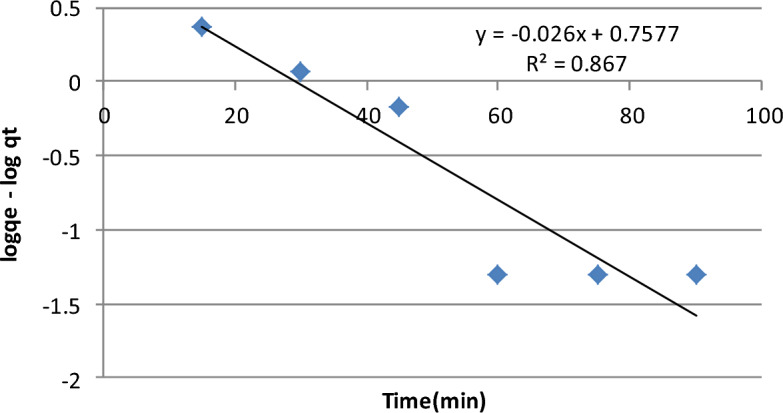
Graphical relation between logqe − logqt and time depicting Pseudo- first-order kinetics of chlorpyrifos—adsorbent (lemon peel) (Optimum condition: Initial concentration = 25 mg L^−1^, Bioadsorbent dose = 1 g, contact time = 60 min pH 3 and temperature = 25 °C).

**Figure 8:**
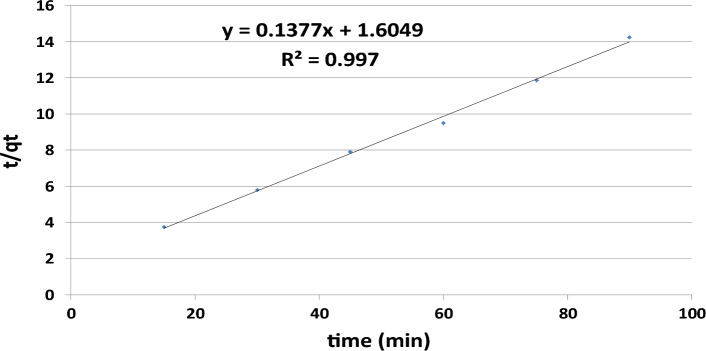
Graphical relation of t/qt against time (min) depicting Pseudo- second-order kinetics of chlorpyrifos—adsorbent (lemon peel) (Optimum condition: Initial concentration = 25 mg L^−1^, Bioadsorbent dose = 1 g, contact time = 60 min pH 3 and temperature = 25 °C).

Pseudo first [Eq. (8)] and second order [Eq. (10)] models were examined for the kinetics investigation^50,51^. From the linearized form of pseudo-first-order kinetics, plotting (Fig. 7) ln (qe – qt) against t gives a linear regression value R^2^ of 0.867. The pseudo-second-order kinetics are given in Fig. 8, which shows the plot of t/q_t_ against t. The linear relationship represents the positive regression value, i.e., R^2^ = 0.997. Modeling data are revealed in Table 2. The determined coefficient value of R^2^ (0.997) is nearly 1 and higher than the pseudo first order model value, hence, the experimental data support the pseudo second order model, as similarly reported by^43,52^. The nonlinear form of kinetics, as performed in many adsorption studies, was not needed for this study. The estimated and experimental Qe values of pseudo-second-order kinetics (7.3 mg g^−1^) and pseudo-first order kinetics (5.71 mg g^−1^) are extremely close to the experimental value of Qe (6.37 mg g^−1^).

**Table 2:** Kinetics data for the experiment.

Kinetic model	Parameters	Calculated values
Pseudo first order	k_1_ (min^−1^)	4.3 × 10^–4^
	Qe (mg g^−1^)	5.71
	R^2^	0.867
Pseudo second order	K_2_ (g mg^−1^ min)	0.0117
	Qe (mg g^−1^)	7.3
	R^2^	0.997
Qe (experimental value)		6.37

### Thermodynamic study of the adsorption of chlorpyrifos on lemon peel

**Figure 9:**
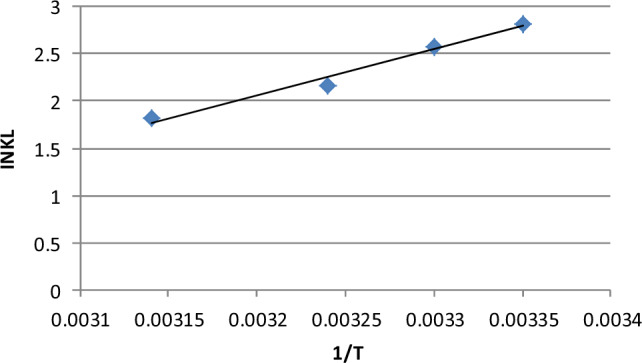
InK_L_ vs 1/T graph for thermodynamic parameters for chlorpyrifos adsorption on lemon peel (Optimum condition: Initial concentration = 25 mg L^−1^, Bioadsorbent dose = 1 g, contact time = 60 min pH 3 and temperature = 25 °C).

The entropy (∆S°), enthalpy (∆H°) and Gibbs free energy (∆G°) values were calculated using a Vant Hoff plot (Fig. 9) for chlorpyrifos adsorption on lemon peel. The nature of the adsorption is determined by the thermodynamic parameters as described by^43,52^. The data are shown in Table 3, and the values of all thermodynamic parameters are less than zero. The values of ∆G° and ∆H° are found to be negative, which indicates that this process is exothermic and that adsorption activity takes place naturally. The negative value of ∆S° shows decreasing randomness at the adsorption interface. The low enthalpy of the adsorption of chlorpyrifos on lemon peel proceeds by physisorption^43^.

**Table 3:** Thermodynamic parameters of adsorption of chlorpyrifos using lemon peel bioadsorbent.

Pesticide	∆H° (kJ mol^−1^)	∆S° (kJ mol K^−1^)	∆G° (kJ mol^−1^)			
			298 K	303 K	308 K	318 K
Chlorpyrifos	− 40.879	− 113.669	− 6.994	− 6.507	− 5.545	− 4.785

### Adsorption equilibrium study using lemon peel

**Figure 10:**
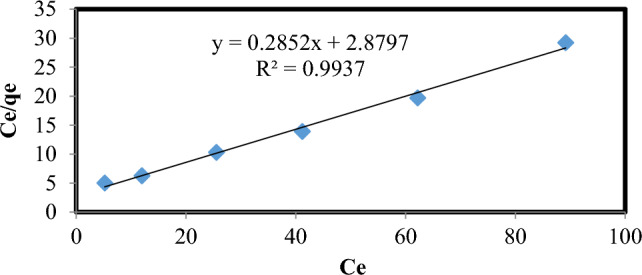
Langmuir isotherm plot of chlorpyrifos adsorbent adsorption system at 298 K (Optimum condition: Initial concentration = 25 mg L^−1^, Bioadsorbent dose = 1 g, contact time = 60 min and pH 3).

**Figure 11:**
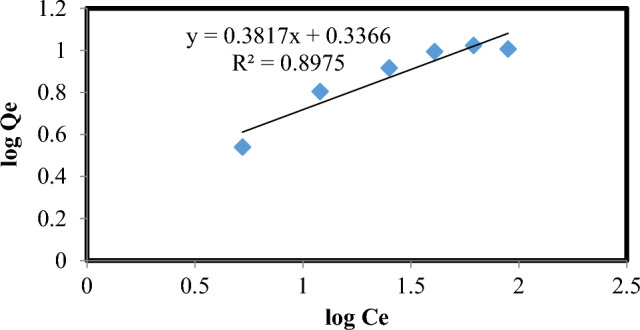
Freundlich isotherm plot of chlorpyrifos adsorption system at 298 K (Optimum condition: Initial concentration = 25 mg L^−1^, Bioadsorbent dose = 1 g, contact time = 60 min and pH 3).

The mechanism of adsorption is described using Freundlich and Langmuir isotherms. The isotherm tests were performed at room temperature with a constant pH of 3. The range of the chlorpyrifos concentration was 10–150 mg/L. Straight line graphs were used to derive the Langmuir and Freundlich parameters. Figures 10 and 11 show the experimental values and model outputs, while Table 4 summarizes the findings. The R^2^ value for the Langmuir isotherm was greater (0.994) than that for the Freundlich isotherm (0.897), and the values of the correlation coefficients for the Langmuir isotherm were higher. Hence, it is observed that the equilibrium data were extremely well matched to the Langmuir isotherm. The monolayer coverage of chlorpyrifos onto adsorption was anticipated by the Langmuir isotherm expression's best fit of equilibrium data, with a maximum sorption capacity of 80%. A favorable adsorption of chlorpyrifos onto lemon peel bioadsorbent is indicated by values of R_L_ for various starting concentrations ranging from 0.04 to 0.24.

**Table 4:** Freundlich and Langmuir isotherm parameters of the chlorpyrifos adsorption system.

Adsorbate	Langmuir isotherm			Freundlich isotherm		
	K_L_	Qmax	R^2^	K_f_	1/n	R^2^
Chlorpyrifos	0.1323	2.624	0.993	2.17	0.381	0.897

### Characterization of lemon peel

**Figure 12:**
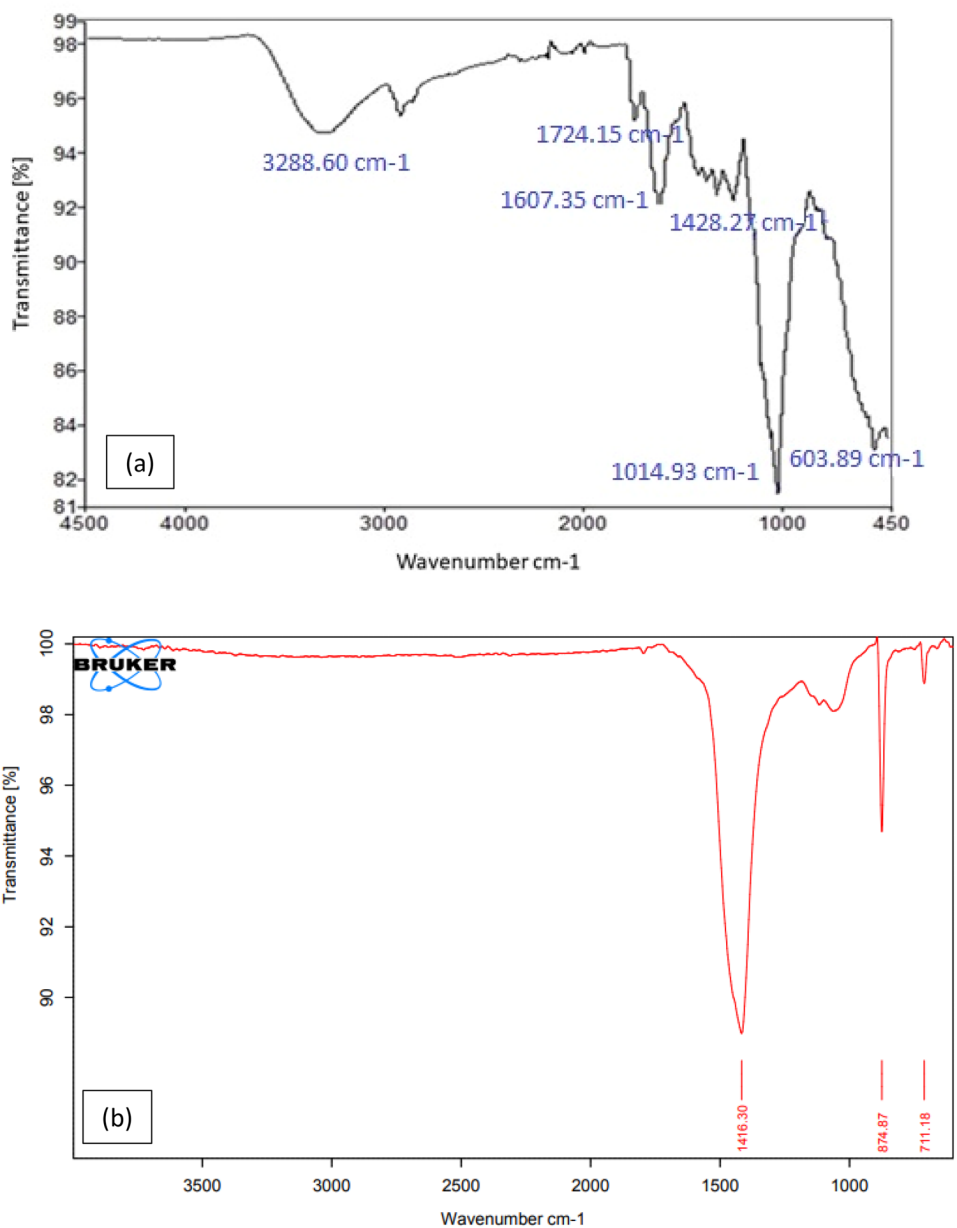
FTIR spectrum of lemon peel (**a**) before, (**b**) after adsorption of chlorpyrifos.

The FTIR data shown in Fig. 12 represent before and after adsorption of chlorpyrifos on the lemon peel bioadsorbent, where some slight changes occur in peak frequencies and a few peaks disappear after sorption. The strong band at 1428.27 cm^−1^, which was assigned to C–H bending in plane vibration, shifted to 1416.30 cm^–1^, which was attributed to O–H bending due to chemical changes that occurred with adsorbed lemon peel. This absorption frequency resulted from the O–H in plane coupling with the C–H wagging vibration in primary and secondary alcohols. A sharp peak was found in the region of 874.87 cm^−1^, which suggests a phosphorus compound due to P–O–P stretching. Mono-substituted benzene was recognized by the band at 711.18 cm^−1^. The FTIR data clearly suggest that adsorption occurred with the lemon peel bioadsorbent.

**Figure 13:**
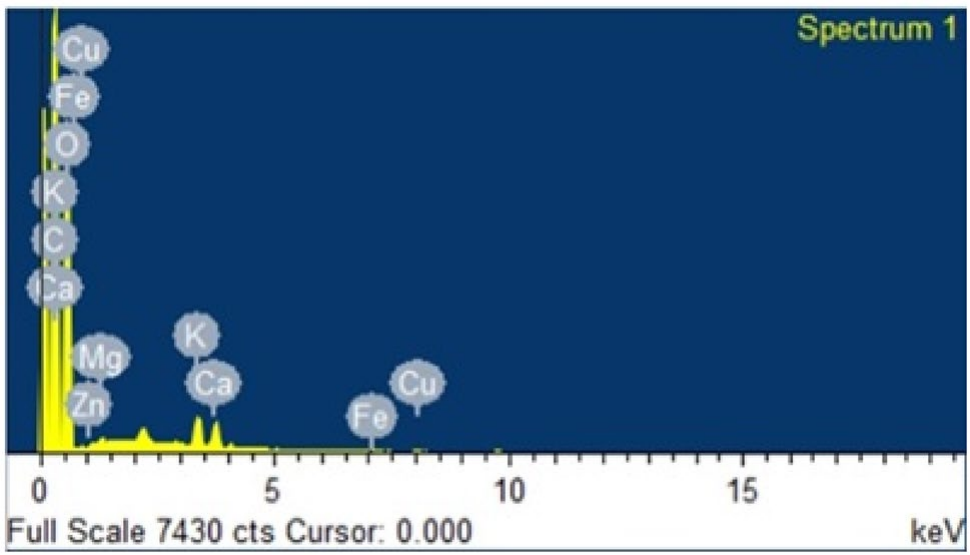
EDX spectrum of lemon peel before adsorption.

**Figure 14:**
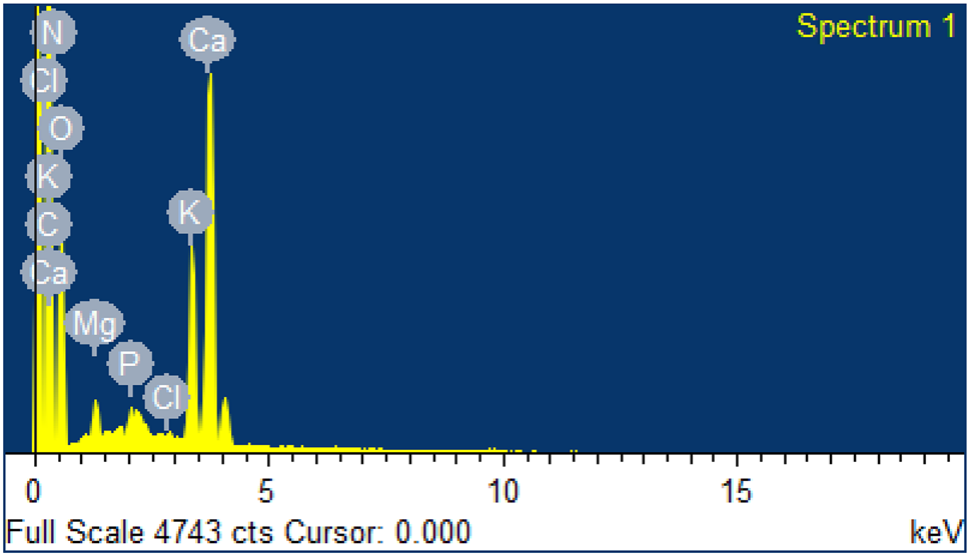
EDX spectrum of lemon peel after adsorption.

**Figure 15:**
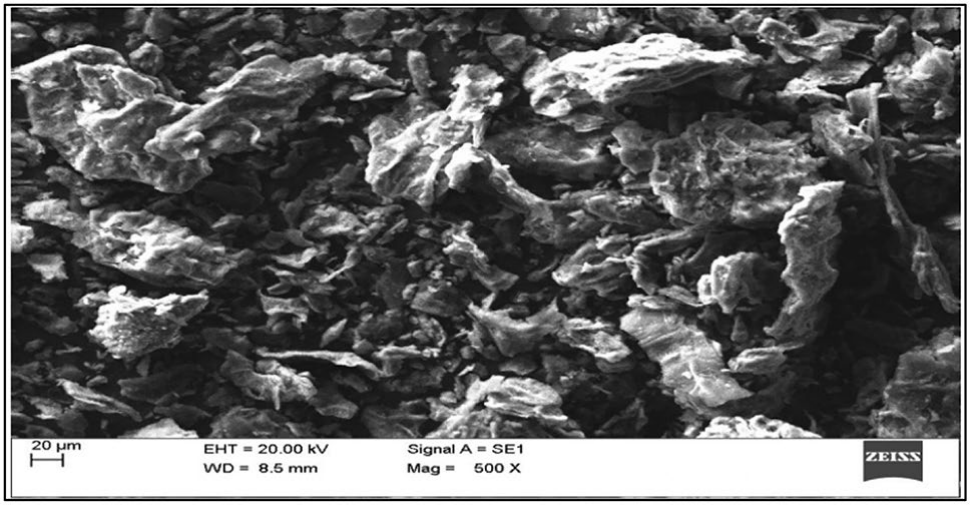
SEM images of lemon peel before adsorption.

**Figure 16:**
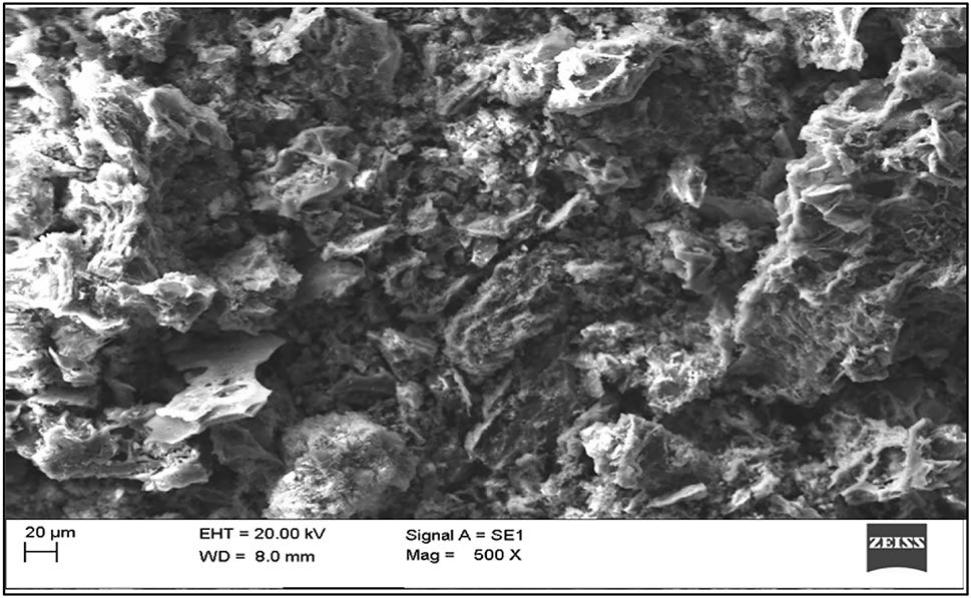
Scanning electron spectrums of lemon peel biochar after adsorption of chlorpyrifos.

The EDX spectra of lemon peel before (Fig. 13) and after (Fig. 14) adsorption indicate high contents of carbon (16.68% and 10.64%) and oxygen (75.66% and 54.87%). SEM images (Figs. 15 and 16) also show the adsorption of chlorpyrifos. The weight percentages of these contents decreased because biomass was pyrolyzed in nature. The main difference found in the spectra before and after adsorption is due to phosphorus and chlorine, which were not available before adsorption but appeared after adsorption in small amounts of 0.60% and 0.26%, respectively, indicating the adsorption of organophosphorus insecticides on lemon peel. Many functional groups contain carbon and oxygen, which are responsible for the adsorption of organic contaminants. These results are also accompanied by FTIR spectra. Potassium in plant biomass remains in the form of cations where organic acids and other anionic groups are neutralized. Magnesium and calcium were found in ionic form as divalent cations. Copper and iron were performed similarly in redox enzymes and allowed electron transfer reactions accompanied by valence changes from Cu^+^ to Cu^2+^. Phosphorus may be present in the form of phosphate anions. These elements in ionic form may be responsible for different types of bioadsorbent surfaces in adsorption. The morphology of the surface was changed with lemon peel loading. The lemon peel surface was more reticulate than before adsorption. It is suggested that chemical changes occurred significantly on the surface of the adsorbed lemon peel. Bioadsorbents derived from lemon peel showed smaller cavities and pores after adsorption. While comparing the pre- and post-adsorption images, multiple deposition patterns were found, which represented the deposition of chlorpyrifos on lemon peel.

### Probable mechanism

The entire adsorption process of chlorpyrifos (organophosphate) adsorption capability and adsorbent surface charge was substantially impacted by pH^53^. FTIR and EDX examination of lemon peel revealed a sufficient amount of nitrogen- and oxygen-containing functional groups such as amine, phenol, and –COOH, with the –COOH group contributing significantly to the adsorbent's negative surface charge^44^. The surface negative potential of bioadsorbents decreases as the pH increases^54^. Hence, the increased electrostatic anion repulsion between negatively charged PO_3_ = S and the bioadsorbent at high solution pH may be the reason for the biosorbent's low affinity for PO_3_ = S. The negatively charged PO_3_ = S ions on bioadsorbent surfaces, as well as the OH– ions, heightened competitive action. The probable mechanism is confirmed by the pH effect on the adsorption mechanism, with the highest adsorption efficiency found at 3 pH attachment is thought to be possible via interactions between oxygen- and nitrogen-containing functional groups present in chlorpyrifos and benzene electron clouds, as well as via hydrogen bonding between an electronegative ion and hydrogen, where the donor ion(o) of chlorpyrifos bonds to the acceptor -OH ion of the biosorbent^55,56^. The notable changes observed in the FTIR spectra include the shift of the band at 1428.27 cm^−1^ to 1416.30 cm^−1^, which is assigned to the OH bending vibration, and a strong band reported at 711.18 cm^−1^, which is a monosubstituted benzene ring. It may be concluded that the adsorption of chlorpyrifos on the lemon peel adsorbent occurs via electrostatic interactions, π-π interactions and hydrogen bonding.

### Comparison of the effectiveness of the study

The experimental results demonstrate that the removal effectiveness ranged from 53 to 77%, which is neither low nor high when compared to other removal reports published globally. Table 5 provides the comparative database, which demonstrates that many of the modified materials are adopted, which would require extremely specialized circumstances to synthesize and utilize. However, the current study shows natural bioadsorbents, which is still significant because the best adsorption occurs with lemon peel 77%, and the quantity of lemon production each year is very high in India. India is ranked first in the production of this fruit, and management of the fruit peels can also be addressed with this approach. Because chlorpyrifos is frequently used in agriculture, industrial, and domestic applications, lemon peel would be an excellent option for adsorbing chlorpyrifos from agricultural flow and water bodies on a broad scale in an inexpensive and environmentally friendly manner.

**Table 5:** Comparative database for removal efficiency of chlorpyrifos with specific conditions based on reported studies worldwide.

Adsorbing materials	Best applied isotherms	Maximum removal efficiency (%) or/and bioadsorbent capacity (mg g^−1^)	Optimum condition	References
Organic residues (composted sewage sludge = RO1, chicken manure = RO2; and a residue from olive oil production called ‘orujillo’ = RO3)	Freundlich isotherm	RO3 > RO2 > RO1 > soil	Concentration = 50–1000 µg L^−1^, contact time = 24 h, pH 7.74	Rojas et al.^57^
Organic substances (sunflower seed shells, rice husk, composted sewage sludge and agricultural soil)	Langmuir isotherm, Pseudo-Second Order	Adsorption capacity 36–67% (rice husk > sunflower seed shells > composted sewage sludge > agricultural soil)	Concentration = 200 µg L^−1^, Adsorbent dosage = 0.6 g, contact time = 20 min/4 h, pH 7.74	Rojas et al.^58^
Biochar, Cyperus alternifolius	–	99%	Concentration = 100 mg L^−1^, Adsorbent dosage = 100 g, contact time = 12 h	Tang et al.^59^
Graphene-based tetraethoxysilane-methyltrimethoxysilane sol–gel hybrid magnetic nano composite (Fe_3_O_4_@G-TEOS-MTMOS)	Langmuir isotherm	> 80%, 37.18 mg g^−1^	Concentration = 25 μg mL^−1^, Adsorbent dosage = 80 mg, contact time = 3 min, pH 6–7	Nodeh et al.^60^
Mesoporous magnesium ferrite (MgFe_2_O_4_)	Langmuir isotherm, Pseudo-Second Order	4200–4460 mg g^−1^	Concentration = 5–60 mg L^−1^, Adsorbent dosage = 0.5 mg, contact time = 50 min, pH 3.5	Sharma and Kakkar^61^
Prussian blue nanorods	Langmuir isotherm, Pseudo-First Order	92.00%	Concentration = 50 mg L^−1^, Adsorbent dosage = 25 mg, contact time = 4 h, pH 7	Rani and shanker^62^
Fe_3_O_4_@SiO_2_@GO-PEA	Langmuir isotherm, Pseudo-Second Order	32.6 mg g^−1^	Temperature = 25 °C, Concentration = 5 µg mL^−1^, Adsorbent dosage = 1.5 mg mL^−1^, contact time = 15 min, pH 7	Wanjeri et al.^63^
Microextraction with magnetically modified graphene	–	< 50%	Concentration = 2 μg mL^−1^, Adsorbent dosage = 20 mg, contact time = 12 min, pH 6	Madej et al.^64^
Polymerization using acrylic acid as monomer	Freundlich isotherm, Pseudo-Second Order	86.23–92.04% and 89.42–102.36%	Concentration = 50 μg mL^−1^, Adsorbent dosage = 20 mg, contact time = 2 h, pH 4	Kumar et al.^65^
Mixed hemimicelle SDS-coated magnetic chitosan nanoparticles (MHMS-MCNPs)	Langmuir isotherm	96%	Temperature = 25 °C, Concentration = 5 ng mL^−1^, contact time = 3 min, pH 2.5	Bandforuzi and Hadjmohammadi^66^
Pristine, malic acid-aged, deashed, and citric acid-aged biochars	Langmuir isotherm	88.7%, 14.8 mg g^−1^	Temperature = 600 °C, Concentration = 3.2 mg L^−1^, Adsorbent dosage = 0.5–1 mg, contact time = 48 h, pH 6.5	Zheng et al.^67^
Biochar, bagasse	Freundlich isotherm, Pseudo-Second Order	89%, 3.20 mg g^−1^	Temperature = 20–35 °C, Concentration = 10 μg L^−1^, Adsorbent dosage = 0.5 g, contact time = 60 min, pH 6.78	Jacob et al.^43^
Hydrogel nanocomposite Arabic Gum-grafted-polyamidoxime and CuFe_2_O_4_ magnetic nanoparticles (AG-g-PAO/CuFe_2_O_4_)	Freundlich isotherm	90%, 270 mg g^−1^	Temperature = 19 °C, Concentration = 0.05 to 1 mg L^−1^, Adsorbent dosage = 0.5 g, contact time = 120 min	Romero et al.^68^
Magnetic adsorbent prepared with hydrocalumite-iron oxide (HC/Fe) modified with dodecyl sulfate (DS)	Langmuir isotherm	72.9 mg g^−1^	Concentration = 25 mg L^−1^, Adsorbent dosage = 1.0 g L^−1^, contact time = 210 min, pH = No change (4–11)	Milagres et al.^69^
Pea peels	Langmuir/Temkin isotherm, Pseudo-Second Order	47.846 mg g^−1^, < 70%	Concentration = 30 µg mL^−1^, Adsorbent dosage = 0.03 g, contact time = 60 min, pH 2	Haq et al.^70^
Mesoporous graphene oxide@zinc oxide (rGO@ZnO)	Freundlich isotherm, Pseudo-Second Order	95.40%	Concentration = 10 mg L^−1^, Adsorbent dosage = 2.5 mg, contact time = 70 min, pH = insensitivity	Gulati et al.^26^
Mesoporous polymers with restricted access materials (RAM M-MIPs)	–	> 90%	Concentration = 5–500 µg L^−1^, Adsorbent dosage = 10 mg, contact time = 5 min	Liang et al.^71^
Nanomaterial (PhaCNCs) synthesized with nano cellulose using grafting polyvinylamine	Langmuir isotherm, Pseudo-Second Order	92.72%, 98.116 mg g^−1^	Concentration = 40 mg L^−1^, Adsorbent dosage = 0.57 g L^−1^, contact time = 8.3 h	Yang et al.^72^
white-rot fungus Trametes versicolor	–	90% removal	Concentration = 5 µg L^−1^, time = 0–14 days	Hu et al.^73^
Cupriavidus nantongensis X1T	Langmuir isotherm, Pseudo-Second Order	160.36 mg g^−1^	Concentration = 20 mg L^−1^, Adsorbent dosage = 40 mg, contact time = 3 h	Abdelhameed et al.^74^
Aminoguanidine modified magnetic graphene oxide	Freundlich isotherm, Pseudo-Second Order	90.39%, 85.47 mg g^−1^	Temperature = 20–40 °C, Concentration = 50–60 mg L^−1^, Adsorbent dosage = 10 mg, contact time = 30 min, pH 6.5	Mahdavi et al.^27^
Nanoscale Moringa olivera seeds waste (nMSW)	Langmuir isotherm, Pseudo-First Order	81%, 25 mg g^−1^	Temperature = 25 °C, Concentration = 25 μg mL^−1^, Adsorbent dosage = 0.05 g, contact time = 30 min, pH 7	Hamadeen et al.^55^
Magnetic sporopollenin supported cyanocalixarene (MSP-CyCalix) nanocomposite	Langmuir isotherm, Pseudo-Second Order	> 90%, 13.88 mg g^−1^	Temperature = 25 °C, Concentration = 10 mg L^−1^, Adsorbent dosage = 40 mg, contact time = 30 min	Kamboh et al.^75^
Leaf extract of Azadirachta indica	Langmuir isotherm, Pseudo-First Order	89–91%	Concentration = 2 mg L^−1^, Adsorbent dosage = 10–35 mg, contact time = 6 h, pH 7	Rani et al.^76^
Nanoparticles of water treated residuals (nWTRs) derived from drinking water industry using plant Alexandria	Langmuir isotherm, Pseudo-First Order	92%, 50 mg g^−1^	Concentration = 25 μg mL^−1^, Adsorbent dosage = 0.05 g, contact time = 30 min, pH 7	Hamadeen et al.^56^
Cinnamon sticks activated carbon	Langmuir isotherm, Pseudo-Second Order	12.37 mg g^−1^	Concentration = 20–50 mg L^−1^, Adsorbent dosage = 0.1 g, contact time = 120 min, pH 7	Ettish et al.^77^
Synthesized GO/ZIF-8 composite	Freundlich isotherm	54.34 mg g^−1^ and 103.72, 83%	Concentration = 20 mg L^−1^, Adsorbent dosage = 16/24 mg, contact time = 24 min, pH 7	Nikou et al.^78^
Cu/CuFe_2_O_4_@iron-based metal–organic framework (Cu/CuFe_2_O_4_@MIL-88A(Fe))	Freundlich isotherm, Pseudo-Second Order	(88.3–100.4%)	Concentration = 0.6–300.0 ng mL^−1^, Adsorbent dosage = 10 mg, contact time = 5 min, pH 7	Amini et al.^79^
Biochar, walnut shells	Langmuir isotherm, Pseudo-Second Order	86.64%, 3.536 mg g^−1^	Temperature = 900 °C, concentration = 1000 µg L^−1^, Adsorbent dosage = 250 µg L^−1^, pH 7.07, time = 0–300 min	Tulun et al.^80^
Biochar, Sugarcane bagasse	Freundlich isotherm, Pseudo-Second Order	86%, 6.25 mg g^−1^	Temperature = 20–40 °C, Concentration = 10–50 µg L^−1^, Adsorbent dosage = 0.5 g, contact time = 40 min, pH 10	Jacob et al.^52^
Biochar, potato peel	–	72.06%	Temperature = RT, Concentration = 1346.85 μg mL^−1^, Adsorbent dosage = 1.04 mg mL^−1^, contact time = 24 h, pH 5.04	Singh et al.^81^
Zinc based green nanocomposites using Sapindus mukorossi seed	Langmuir isotherms, Pseudo First Order	92%, 3.57 mg g^−1^	Concentration = 2 mg L^−1^, Adsorbent dosage = 25 mg, contact time = approximately 1 h, pH 7	Yadav et al.^82^
Hydrogel nanocomposite Arabic Gum-grafted-polyamidoxime and CuFe_2_O_4_ magnetic nanoparticles (AG-g-PAO/CuFe_2_O_4_)	Freundlich isotherm, Pseudo-Second Order	769.23 mg g^−1^	Concentration = 50–300 mg L^−1^, Adsorbent dosage = 0.005 g, contact time = 20 min, pH 6	Hassanzadeh-Afruzi et al.^83^
Cotton and synthetic hybrid cotton NH2-UiO-101-Zr@Ox-cotton	Langmuir isotherm, Pseudo-Second Order	389.69 mg g^−1^	Concentration = 25–250 mg L^−1^, Adsorbent dosage = 750 mg L^−1^, contact time = 4 h	Abdelhameed and Emam^84^
Nano structured activated biochar based pomegranate peels (nABPP)	Langmuir isotherm, Pseudo-First Order	90%, 100 mg g^−1^	Concentration = 25 μg mL^−1^, Adsorbent dosage = 0.05–0.5 g, contact time = 10 min, pH 7	Hamadeen et al.^85^
Peels from cassava roots, crambe meal, and Pinus barks	Freundlich isotherm	16 g L^−1^, 99.2%	Concentration = 1000 µg L^−1^, Adsorbent dosage = 4.0 g L^−1^, contact time = 10–20 min, pH 3–7	Schwantes et al.^86^
Chinese cabbages and green onions using metal organic frameworks	–	63.73 to 96.58%	Concentration = 0–100 ng mL^−1^, Adsorbent dosage = 10 mg, pH 10, time = 45 min	Liu et al.^87^
Magnetic graphene oxide and carboxymethyl cellulose (MGOC)	Langmuir isotherm, Pseudo-Second Order	98.3%, 108.3 mg g^−1^	Concentration = 14.0 mg L^−1^, Adsorbent dosage = 0.40 g L^−1^, contact time = 40 min, pH 6.0	Dolatabadi et al.^88^
Lemon peel	Freundlich isotherm, Pseudo-Second Order	77%	Concentration = 3.24–6.43 µg/100 mL0mL, Bioadsorbent dosage = 1 g, contact time = 60 min, pH 3.0	Present Study
Watermelon	Freundlich isotherm, Pseudo-Second Order	67%	Concentration = 3.24–6.43 µg/100 mL, Bioadsorbent dosage = 1 g, contact time = 60 min, pH 3.0	Present study
Guava leaves	Freundlich isotherm, Pseudo-Second Order	61%	Concentration = 3.24–6.43 µg/100 mL, Bioadsorbent dosage = 1 g, contact time = 60 min, pH 3.0	Present study
Cauliflower	Freundlich isotherm, Pseudo-Second Order	56%	Concentration = 3.24–6.43 µg/100 mL, Bioadsorbent dosage = 1 g, contact time = 60 min, pH 3.0	Present study
Bael	Freundlich isotherm, Pseudo-Second Order	53%	Concentration = 3.24–6.43 µg/100 mL, Bioadsorbent dosage = 1 g, contact time = 60 min, pH 3.0	Present study

## Conclusion

Chlorpyrifos is the major cause of environmental pollution, and it is necessary to work on a sustainable approach to solve this issue. A variety of approaches were employed to describe the biomass before it was used as a bioadsorbent to separate and extract small portions of chlorpyrifos from the aqueous media. The removal and extraction efficiency were improved by adjusting factors including pH, bioadsorbent value, adsorption period, temperature, and adsorbate concentration. The findings indicated that pH 3, an absorption time of 60 min, a bioadsorbent value of 1 g, and an initial concentration of 25 mg/L chlorpyrifos, and for real samples, 3.24–6.43 µg/100 mL at room temperature were the ideal parameters for lemon peel in this study. Four other bioadsorbents used in this investigation had comparatively minimal effectiveness against pesticides. The equilibrium data were modeled by two isotherm equations. Among both, the Langmuir isotherm gave a better fit to the sorption equilibrium data according to the R^2^ value, which shows homogeneity of the sorption process. The surface adsorption process aligns with the semi second-order model, as shown by the outcomes of synthetic trials. The analyte's adsorption on the bioadsorbent was thus accomplished through chemical adsorption. The usage of lemon peel would handle two issues: chlorpyrifos containing water and trash fruit, and the procedure will be sustainable. As this study was performed with laboratory-made solutions as well as real samples from various fields, the results of the present work will guide the development of a commercial clean production unit to apply this biomass in the treatment of chlorpyrifos-contaminated water.

## Data Availability

The datasets used and/or analyzed during the current study are available from the corresponding author on reasonable request. Data can be obtained by emailing manojjindal1989@gmail.com or santosh.sar@bitdurg.ac.in.

## References

[1] Hazardous Substances Databank (HSDB). *Chlorpyrifos* (U.S. Department of Health and Human Services, National Institutes of Health, National Library of Medicine, 2005).

[2] USEPA. *Interim Reregistration Eligibility Decision for Chlorpyrifos. Report No EPA738-R-01-007*. (U.S. EPA, 2002).

[3] Grube, D., Donaldson, T. & Wu, L. *Pesticide Industry Sales and Usage—2006 and 2007 Market Estimates. USEPA, Rep. 733-R-11-001, 41* (2011).

[4] Eaton DL (2008). Review of the toxicology of chlorpyrifos with an emphasis on human exposure and neurodevelopment. Crit. Rev. Toxicoogyl..

[5] Gavrilescu M (2005). Fate of pesticides in the environment and its bioremediation. Eng. Life Sci..

[6] Van Loon GW, Duffy SJ (2005). Environmental Chemistry: A Global Perspective.

[7] Mugni, H. N. *et al*. (2012). Toxicity persistence in runoff water and soil in experimental soybean plots following chlorpyrifos application. *Bull. Environ. Contam. Toxicol*. **89**(1), 208–212 (2012). 10.1007/s00128-012-0643-6 22526996

[8] Gebremariam, S. Y., Beutel, M. W., Yonge, D. R., Flury, M. & Harsh, J. B. *Adsorption and Desorption of Chlorpyrifos to Soils and Sediments. Reviews of Environmental Contamination and Toxicology*, 123–175 (Springer, 2012). 10.1007/978-1-4614-1463-6_3 22057931

[9] Liu Y, Li S, Ni Z, Qu M, Zhong D, Ye C (2016). Fubin Tang pesticides in persimmons, jujubes and soil from China: Residue levels, risk assessment and relationship between fruits and soils. Sci. Total Environ..

[10] World Health Organization. *The WHO Recommended Classification of Pesticides by Hazard and Guidelines to Classification* (2009) (Report). (World Health Organization, 2009).

[11] Coupe RH, Manning MA, Foreman WT, Goolsby DA, Majewski MS (2000). Occurrence of pesticides in rain and air in urban and agricultural areas of Mississippi, April–September 1995. Sci. Total Environ..

[12] Gilliom, R. J. *et al*. *The Quality of Our Nation’s Waters: Pesticides in the Nation’s Streams and Ground Water*, 1992–2001. (U.S. Geological Survey, 2006).

[13] Glotfelty DE, Seiber JN, Liljedahl A (1987). Pesticides in fog. Nature.

[14] Hoffman RS, Capel PD, Larson SJ (2000). Comparison of pesticides in eight urban streams. Environ. Toxicol. Chem..

[15] Kolpin DW, Barbash JE, Gilliom RJ (2000). Pesticides in ground water of the United States, 1992–1996. Ground Water.

[16] Kuang Z, McConnell LL, Torrents A, Meritt D, Tobash S (2003). Atmospheric deposition of pesticides to an agricultural watershed of the Chesapeake Bay. J. Environ. Qual..

[17] Readman, J. W. *et al*. Persistent organophosphorus pesticides in tropical marine environments. *Mar. Pollut. Bull*. **24**(8), 398–402 (1992).

[18] Wightwick A, Allinson G (2007). Pesticide residues in victorian waterways: A review. Aust. J. Ecotoxicol..

[19] Zamora, K. C. R., Majewski, M. S. & Knifong, D. L. *Diazinon and Chlorpyrifos Loads in Precipitation and Urban and Agricultural Storm Runoff During January and February 2001 in the San Joaquin River Basin, California: Water-Resource Investigation Report 03-4091*. (U.S. Geological Survey, 2003).

[20] Rahman HU, Asghar W, Nazir W, Sandhu MA, Ahmed A, Khalid N (2021). A comprehensive review on chlorpyrifos toxicity with special reference to endocrine disruption: Evidence of mechanisms, exposures and mitigation strategies. Sci. Total Environ..

[21] Morgan MK, Sheldon LS, Croghan CW, Jones PA, Robertson GL, Chuang JC, Wilson NK, Lyu CW (2005). Exposures of preschool children to chlorpyrifos and its degradation product 3, 5, 6-trichloro-2-pyridinol in their everyday environments. J. Eposure Sci. Environ. Epidemiol..

[22] Guo J, Zhang J, Wu C, Lv S, Lu D, Qi X, Jiang S, Feng C, Yu H, Liang W, Chang X (2019). Associations of prenatal and childhood chlorpyrifos exposure with neurodevelopment of 3-year-old children. Environ. Pollut..

[23] Wang JL, Xu LJ (2012). Advanced oxidation processes for wastewater treatment formation of hydroxyl radical and application. Crit. Rev. Environ. Sci. Technol..

[24] Hossain MS, Fakhruddin ANM, Chowdhury MAZ, Alam MK (2013). Degradation of chlorpyrifos, an organophosphorus insecticide in aqueous solution with gamma irradiation and natural sunlight. J. Environ. Chem. Eng..

[25] Affam C, Chaudhuri M, Kutty SRM, Muda K (2014). UV Fenton and sequencing batch reactor treatment of chlorpyrifos, cypermethrin and chlorothalonil pesticide wastewater. Int. Biodeterior. Biodegrad..

[26] Gulati A, Malik J, Kakkar R (2020). Mesoporous RGO@ZnO composite: Facile synthesis and excellent water treatment performance by pesticide adsorption and catalytic oxidative dye degradation. Chem. Eng. Res. Des..

[27] Mahdavi V. *et al*. Aminoguanidine modified magnetic graphene oxide as a robust nanoadsorbent for efficient removal and extraction of chlorpyrifos residue from water. *J. Environ. Chem. Eng*. **9**, 106177. 10.1016/j.jece.2021.106117 (2021).

[28] Deshmukh P, Sar SK, Jindal MK (2023). Plant mediated magnetic nano composite as promising scavenger's radionuclides for the efficient remediation in aqueous medium. Chemosphere.

[29] Deshmukh P, Sar SK, Jindal MKR (2023). Magnetite based green bio composite for uranium exclusion from aqueous solution. J. Radioanal. Nucl. Chem..

[30] Saleh TA, Tuzen M, Sarı A (2017). Magnetic activated carbon loaded with tungsten oxide nanoparticles for aluminum removal from waters. J. Environ. Chem. Eng..

[31] Saleh TA, Tuzen M, Sarı A (2017). Polyethylenimine modified activated carbon as novel magnetic adsorbent for the removal of uranium from aqueous solution. Chem. Eng. Res. Des..

[32] Tuzen M, Sarı A, Saleh TA (2018). Response surface optimization, kinetic and thermodynamic studies for effective removal of rhodamine B by magnetic AC/CeO_2_ nanocomposite. J. Environ. Manage..

[33] Altıntıg E, Altundag H, Tuzen M, Sarı A (2017). Effective removal of methylene blue from aqueous solutions using magnetic loaded activated carbon as novel adsorbent. Chem. Eng. Res. Des..

[34] Altintig E, Onaran M, Sarı A, Altundag H, Tuzen M (2018). Preparation, characterization and evaluation of bio-based magnetic activated carbon for effective adsorption of malachite green from aqueous solution. Mater. Chem. Phys..

[35] Majors, R. E. Solid-phase extraction. in *Handbook of Sample Preparation* (eds Pawliszyn, J. & Lord, H. L.). (Springer, 2010).

[36] Hameed K, Chai D, Rassau A (2018). A comprehensive review of fruit and vegetable classification techniques. Image Vis. Comput..

[37] Kovačević, D. B. *et al*. *Chapter 2: Strategies to Achieve a Healthy and Balanced Diet: Fruits and Vegetables as a Natural Source of Bioactive Compounds*. *Agri-Food Industry Stratgies for Healthy Diet and Sustainiblity*, 51–88 (2020).

[38] Lakshmipathy R, Sarada NC (2013). Application of watermelon rind as sorbent for removal of nickel and cobalt from aqueous solution. Int. J. Miner. Process..

[39] Chakravarty S, Mohanty A, Sudha TN, Upadhyay AK, Konar J, Sircar JK, Madhukar A, Gupta KK (2010). Removal of Pb(II) ions from aqueous solution by adsorption using bael leaves (*Aegle marmelos*). J. Hazard Mater..

[40] Ponnusami V, Vikram S, Srivastava SN (2008). Guava (*Psidium guajava*) leaf powder: Novel adsorbent for removal of methylene blue from aqueous solutions. J. Hazard. Mater..

[41] Ponnuchamy M, Kapoor A, Sivaraman P, Ramasamy K (2020). Environmental science and pollution. Research.

[42] APEDA data base, Agricultural & Processed Food Products Export Development Authority (Ministry of Commerce & Industry, Govt. of India). https://agriexchange.apeda.gov.in/International_Productions/International_Production.aspx?ProductCode=0497.

[43] Jacob MM, Ponnuchamy M, Kapoor A, Sivaraman P (2020). Bagasse based biochar for the adsorptive removal of chlorpyrifos from contaminated water. J. Environ. Chem. Eng..

[44] Joshi V, Chhatterji J, Sar SK (2020). Characterization of plant based low cost adsorbents in Chhattisgarh state. Indian J. Ecol..

[45] Rane NM, Admane SV, Sapkal RS (2019). Adsorption of hexavalent chromium from wastewater by using sweetlime and lemon peel powder by batch studies. Waste Manag. Resour. Effic..

[46] Nandhini AR, Harshiny M, Gummadi SN (2021). Chlorpyrifos in environment and foods: A critical review of detection methods and degradation pathways. Environ. Sci..

[47] Nallapaneni, A. & Pope, C. N. Chlorpyrifos. in *Encyclopedia of Toxicology*, 2nd edn, 583–585 (ed Wexler, P.) (Elsevier, 2005).

[48] Liu HF, Ku CH, Chang SS, Chang CM, Wang IK, Yang HY, Weng CH, Huang WH, Hsu CW, Yen TH (2020). Outcome of patients with chlorpyrifos intoxication. Hum. Exp. Toxicol..

[49] Rathod AL, Garg RK (2017). Chlorpyrifos poisoning and its implications in human fatal cases: A forensic perspective with reference to Indian scenario. J. Forensic Leg. Med..

[50] Blanchard G, Maunaye M, Martin G (1984). Removal of heavy metals from waters by means of natural zeolites. Water Res..

[51] Lagergren, S. Zur theorie der sogenannten adsorption geloster Stoffe. *Z. Chem. Ind. Kolloide*. **2**, 15. 10.1007/BF01501332(1907).

[52] Jacob MM, Ponnuchamy M, Kapoor A, Sivaraman P (2022). Adsorptive decontamination of organophosphate pesticide chlorpyrifos from aqueous systems using bagasse-derived biochar alginate beads: thermodynamic, equilibrium, and kinetic studies. Chem. Eng. Res. Des..

[53] Eduah JO, Nartey EK, Abekoe MK, Henriksen SW, Andersen MN (2020). Mechanism of orthophosphate (PO_4_-P) adsorption onto different biochars. Environ. Technol. Innov..

[54] Li H, Dong X, da Silva EB, de Oliveira LM, Chen Y, Ma LQ (2017). Mechanisms of metal sorption by biochars: Biochar characteristics and modifications. Chemosphere.

[55] Hamadeen HM, Elkhatib EA, Badawy MEI, Abdelgaleil SAM (2021). Novel low cost nanoparticles for enhanced removal of chlorpyrifos from wastewater: Sorption kinetics, and mechanistic studies. Arab. J. Chem..

[56] Hamadeen HM, Elkhatib EA, Badawy ME, Abdelgaleil SA (2021). Green low cost nanomaterial produced from *Moringa oleifera* seed waste for enhanced removal of chlorpyrifos from wastewater: Mechanism and sorption studies. J. Environ. Chem. Eng..

[57] Rojas R, Morillo J, Usero J, Delgado-Moreno L, Gan J (2013). Enhancing soil sorption capacity of an agricultural soil by addition of three different organic wastes. Sci. Total Environ..

[58] Rojas R, Morillo J, Usero J, Vanderlinden E, El Bakouri H (2015). Adsorption study of low-cost and locally available organic substances and a soil to remove pesticides from aqueous solutions. J. Hydrol..

[59] Tang X, Yang Y, Tao R, Chen P, Dai Y, Jin C, Feng X (2016). Fate of mixed pesticides in an integrated recirculating constructed wetland (IRCW). Sci. Total Environ..

[60] Nodeh Rashidi, H., Ibrahim, W. A. W., Kamboh, M. A. & Sanagi, M. M. New magnetic graphene-based inorganic–organic sol-gel hybrid nanocomposite for simultaneous analysis of polar and non-polar organophosphorus pesticides from water samples using solid-phase extraction. *Chemosphere*. **166**, 21–30. 10.1016/j.chemosphere.2016.09.054 (2017). 10.1016/j.chemosphere.2016.09.054 27681257

[61] Sharma L, Kakkar R (2018). Magnetically retrievable one-pot fabrication of mesoporous magnesium ferrite (MgFe_2_O_4_) for the remediation of chlorpyrifos and real pesticide wastewater. J. Environ. Chem. Eng..

[62] Rani M, Shanker U (2018). Effective adsorption and enhanced degradation of various pesticides from aqueous solution by Prussian blue nanorods. J. Environ. Chem. Eng..

[63] Wanjeri VWO, Sheppard CJ, Prinsloo ARE, Ngila JC, Ndungu PG (2018). Isotherm and kinetic investigations on the adsorption of organophosphorus pesticides on graphene oxide based silica coated magnetic nanoparticles functionalized with 2-phenylethylamine. J. Environ. Chem. Eng..

[64] Madej K, Jonda A, Borcuch A, Piekoszewski W, Chmielarz L, Gil B (2019). A novel stir bar sorptive-dispersive microextraction in combination with magnetically modified graphene for isolation of seven pesticides from water samples. Microchem. J..

[65] Kumar N, Narayanan N, Gupta S (2019). Ultrasonication assisted extraction of chlorpyrifos from honey and brinjal using magnetic molecularly imprinted polymers followed by GLC-ECD analysis. React. Funct. Polym..

[66] Bandforuzi SR, Hadjmohammadi MR (2019). Modified magnetic chitosan nanoparticles based on mixed hemimicelle of sodium dodecyl sulfate for enhanced removal and trace determination of three organophosphorus pesticides from natural waters. Anal. Chim. Acta.

[67] Zheng H, Zhang Q, Liu G, Luo X, Li F, Zhang Y, Wang Z (2019). Characteristics and mechanisms of chlorpyrifos and chlorpyrifos-methyl adsorption onto biochars: Influence of deashing and low molecular weight organic acid (LMWOA) aging and co-existence. Sci. Total Environ..

[68] Romero, V. *et al*. Efficient adsorption of endocrine-disrupting pesticides from water with a reusable magnetic covalent organic framework. *Microporous Mesoporous Mater*. **307**, 1110523. 10.1016/j.micromeso.2020.110523 (2020).

[69] Milagres, J. L., Bellato, C. R., Ferreira, S. O. & De Moura Guimarães, L. Preparation and evaluation of hydrocalumite-iron oxide magnetic intercalated with dodecyl sulfate for removal of agrichemicals. *J. Environ. Manag*. **255**, 109845. 10.1016/j.jenvman.2019.109845 (2020). 10.1016/j.jenvman.2019.109845 31778866

[70] Haq A, Saeed M, Usman M, Naqvi SAR, Bokhari TH, Maqbool T, Ghaus H, Tahir T, Khalid H (2020). Sorption of chlorpyrifos onto zinc oxide nanoparticles impregnated pea peels (*Pisum sativum* L.): Equilibrium, kinetic and thermodynamic studies. Environ. Technol. Innov..

[71] Liang, T., Chen, L. & Ma, Y. Mesoporous structured molecularly imprinted polymer with restricted access function for highly selective extraction of chlorpyrifos from soil. *J. Chromatogr. A*. **1609**, 460453. 10.1016/j.chroma.2019.460453 (2020). 10.1016/j.chroma.2019.460453 31445801

[72] Yang, J. *et al*. Optimization of polyvinylamine-modified nanocellulose for chlorpyrifos adsorption by central composite design. *Carbohydr. Polym*. 10.1016/j.carbpol.2020.116542 (2020). 10.1016/j.carbpol.2020.116542 32718637

[73] Hu, K. *et al*. Exploring the degradation capability of trametes versicolor on selected hydrophobic pesticides through setting sights simultaneously on culture broth and biological matrix. *Chemosphere*. **250**, 126293. 10.1016/j.chemosphere.2020.126293 (2020). 10.1016/j.chemosphere.2020.126293 32234621

[74] Abdelhameed RM, Shaltout AA, Mahmoud MHH, Emam HE (2021). Efficient elimination of chlorpyrifos via tailored macroporous membrane based on Al-MOF. Sustain. Mater. Technol..

[75] Kamboh MA, Arain SS, Jatoi AH, Sherino B, Algarni TS, Al-onazi WA, Al-Mohaimeed AM, Rezania S (2021). Green sporopollenin supported cyanocalixarene based magnetic adsorbent for pesticides removal from water: Kinetic and equilibrium studies. Environ. Res..

[76] Rani M, Yadav J, Shanker U (2021). Green synthesis of sunlight responsive zinc oxide coupled cadmium sulfide nanostructures for efficient photodegradation of pesticides. J. Colloid Interface Sci..

[77] Ettish, M. N., El-Sayyad, G. S., Elsayed, M. A. & O. Abuzalat. Preparation and characterization of new adsorbent from cinnamon waste by physical activation for removal of chlorpyrifos. *Environ. Chall*. **5**, 100208. 10.1016/j.envc.2021.100208 (2021).

[78] Nikou, M., Samadi-Maybodi, A., Yasrebi, K., & Sedighi-Pashaki, E. Simultaneous monitoring of the adsorption process of two organophosphorus pesticides by employing GO/ZIF-8 composite as an adsorbent. *Environ. Technol. Innov*. **23**, 101590. 10.1016/j.eti.2021.101590 (2021).

[79] Amini S, Amiri M, Ebrahimzadeh H, Seidi S, Kandeh SH (2021). Synthesis of magnetic Cu/CuFe_2_O_4_@MIL-88A(Fe) nanocomposite and application to dispersive solid-phase extraction of chlorpyrifos and phosalone in water and food samples. J. Food Compos. Anal..

[80] Tulun, Ş., Akgül, G., Alver, A. & Çelebi, H. Adaptive neuro-fuzzy interference system modelling for chlorpyrifos removal with walnut shell biochar. *Arab. J. Chem.* **14**, 12. 10.1016/j.arabjc.2021.103443 (2021).

[81] Singh, M. *et al*. Characterization of organophosphate pesticide sorption of potato peel biochar as low cost adsorbent for chlorpyrifos removal. *Chemosphere*. **297**, 134112. 10.1016/j.chemosphere.2022.134112 (2022). 10.1016/j.chemosphere.2022.134112 35227752

[82] Yadav, J., Rani, M. & Shanker, U. An integrated hybrid nanoplatform with polymer coating: Zinc based green nanocomposites with improved photoactivity under sunlight irradiation. *J. Environ. Chem. Eng*. **10**, 3. 10.1016/j.jece.2022.107452 (2022).

[83] Hassanzadeh-Afruzi F, Maleki A, Zare EN (2022). Efficient remediation of chlorpyrifos pesticide from contaminated water by superparamagnetic adsorbent based on Arabic gum-grafted-polyamidoxime. Int. J. Biol. Macromol..

[84] Abdelhameed RM, Emam HE (2022). Modulation of metal organic framework hybrid cotton for efficient sweeping of dyes and pesticides from wastewater. Sustain. Mater. Technol..

[85] Hamadeen, H. M. & Elkhatib, E. A. Nanostructured modified biochar for effective elimination of chlorpyrifos from wastewater: Enhancement, mechanisms and performance. *J. Water Process Eng.* **47**, 102703. 10.1016/j.jwpe.2022.102703 (2022).

[86] Schwantes D, Gonçalves AC, Fuentealba D, Carneiro MFH, Tarley CRT, Prete MC (2022). Removal of chlorpyrifos from water using biosorbents derived from Cassava Peel, Crambe Meal, and Pinus Bark. Chem. Eng. Res. Des..

[87] Liu, G. *et al*. Adsorption and removal of organophosphorus pesticides from Chinese cabbages and green onions by using metal organic frameworks based on the mussel-inspired adhesive interface. *Food Chem. 393*, 133337. 10.1016/j.foodchem.2022.133337 (2022). 10.1016/j.foodchem.2022.133337 35653990

[88] Dolatabadi M, Naidu H, Ahmadzadeh S (2022). Adsorption characteristics in the removal of chlorpyrifos from groundwater using magnetic graphene oxide and carboxy methyl cellulose composite. Sep. Purif. Technol..

